# EZH2 promotes angiogenesis through inhibition of miR-1/Endothelin-1 axis in nasopharyngeal carcinoma

**DOI:** 10.18632/oncotarget.2435

**Published:** 2014-09-03

**Authors:** Juan Lu, Fei-Peng Zhao, Zengliu Peng, Meng-Wen Zhang, Shao-Xiong Lin, Bi-Jun Liang, Bao Zhang, Xiong Liu, Lu Wang, Gang Li, Wen-Dong Tian, Ying Peng, Ming-Liang He, Xiang-Ping Li

**Affiliations:** ^1^ Department of Otolaryngology-Head and Neck Surgery, Nanfang Hospital, Southern Medical University, Guangzhou, China; ^2^ Lab of Otolaryngology & Head and Neck tumor, Nanfang Hospital, Southern Medical University, Guangzhou, China; ^3^ Department of Otolaryngology-Head and Neck Surgery, The First Affiliated Hospital, Shantou University Medical College, Shantou, China; ^4^ School of Public Health and Tropical Medicine, Southern Medical University, Guangzhou, China; ^5^ Department of Neurology, The Sun Yat-sen Memorial Hospital, Sun Yat-sen University, Guangzhou, China; ^6^ Key Laboratory of malignant tumor gene regulation and target therapy of Guangdong Higher Education Institutes, Sun Yat-sen University, Guangzhou, China; ^7^ Department of Biomedical Science, City University of Hong Kong, Hong Kong, China

**Keywords:** EZH2, miR-1, Endothelin-1, angiogenesis, nasopharyngeal carcinoma

## Abstract

Emerging evidence clearly indicates that EZH2 plays a crucial role in tumor angiogenesis. However, the role of EZH2 in angiogenesis is still unknown in nasopharyngeal carcinoma (NPC). We here showed that the elevated EZH2 level was closely associated with an aggressive and poor prognostic phenotype, and was positively correlated with microvessel density (MVD) in NPC tissues. Functional studies showed that EZH2 upregulation promoted cell proliferation, migration and tubule formation of endothelial cells, and knockdown of EZH2 suppressed tumor growth, metastasis and angiogenesis *in vivo*. Mechanistic investigations revealed that EZH2 inhibited miR-1 transcription via promoter binding activity, leading to enhanced expression of Endothelin-1 (ET-1) which is suppressed by miR-1 targeting of ET-1 3′UTR. Furthermore, knockdown of EZH2 or overexpression of miR-1 exerted anti-angiogenic effect on NPC cells. More importantly, the neutralizing antibody against ET-1 significantly abrogated the pro-angiogenic effect of EZH2, and forced expression of ET-1 rescued the anti-angiogenic effect induced by EZH2 knockdown. In clinical specimens, ET-1 was widely overexpressed and associated with clinical stage and MVD. Taken together, our results identify a novel signaling pathway involved in NPC angiogenesis, and also suggest that EZH2-miR-1-ET-1 axis represents multiple potential therapeutic targets for NPC.

## INTRODUCTION

Nasopharyngeal carcinoma (NPC) is a remarkably unique type of head and neck squamous cell carcinomas, which is highly prevalent in Southern China. The primary tumor usually arises from the lateral walls of the nasopharynx, and is characterized by a rich submucosal network of lymphatics, often leading to cervical lymph node metastasis [1]. Distant metastasis has been identified as a main cause of treatment failure in patients with NPC. Therefore, better strategies of treatment will ultimately require understanding of the molecular mechanisms of the metastasis steps of NPC and specifically targeting the critical signaling effectors.

Tumor angiogenesis and metastatic spreading are two highly interconnected phenomena, contributing to cancer-associated deaths. Pathological angiogenesis is a hallmark of cancer and is necessary for tumors to keep growing and spreading [2]. This process is regulated by a balance between pro- and anti-angiogenic molecules. Recent evidences identified Endothelin-1 (ET-1) as a potential autocrine regulator of endothelial cells in the different steps of neovascularization, including proliferation, migration, invasion, protease production and morphogenesis [3]. ET-1 can also modulate cancer angiogenesis indirectly through the induction of vascular endothelial growth factor (VEGF) [4, 5]. Therefore, ET-1-induced, VEGF-dependent angiogenesis may be a novel therapeutic strategy for tumor angiogenesis and metastasis.

Enhancer of Zeste Homolog 2 (EZH2) is the catalytic subunit of Polycomb repressive complex 2 (PRC2), which methylates histone H3 lysine 27, thereby silencing multiple tumor suppressor genes [6]. Upregulation of EZH2 in tumor cells, which is common in both hematopoietic malignancies and solid tumors, promotes cell growth, migration and invasion. High level of EZH2 expression predicts poor prognosis, high grade, high stage in multiple cancer types [7]. Recently, a newly identified mechanism has been observed, which proves that EZH2 plays a crucial role in tumor angiogenesis [8]. Our previous studies demonstrated that EZH2 was overexpressed in NPC cell lines and tissues, which promoted tumor growth and metastasis *in vitro* and *in vivo* [9, 10]. These findings were further confirmed by Tong's report, which showed that EZH2 supported NPC aggressiveness by forming a co-repressor complex with HDAC1/HDAC2 and Snail to inhibit E-cadherin expression [11]. However, the role of EZH2 in other steps of the metastatic process, such as tumor angiogenesis, has never been documented in NPC.

In this study, we investigated the potential involvement of EZH2 in tumor angiogenesis of NPC. The results showed that EZH2 promoted angiogenesis *in vitro* and *in vivo*, which was mediated by inhibition of miR-1/ET-1 axis. Our study will provide useful targets to develop novel and more effective anti-angiogenic and anti-metastatic therapy.

## RESULTS

### EZH2 is overexpressed and positively correlated with microvessel density in NPC

**Figure 1:**
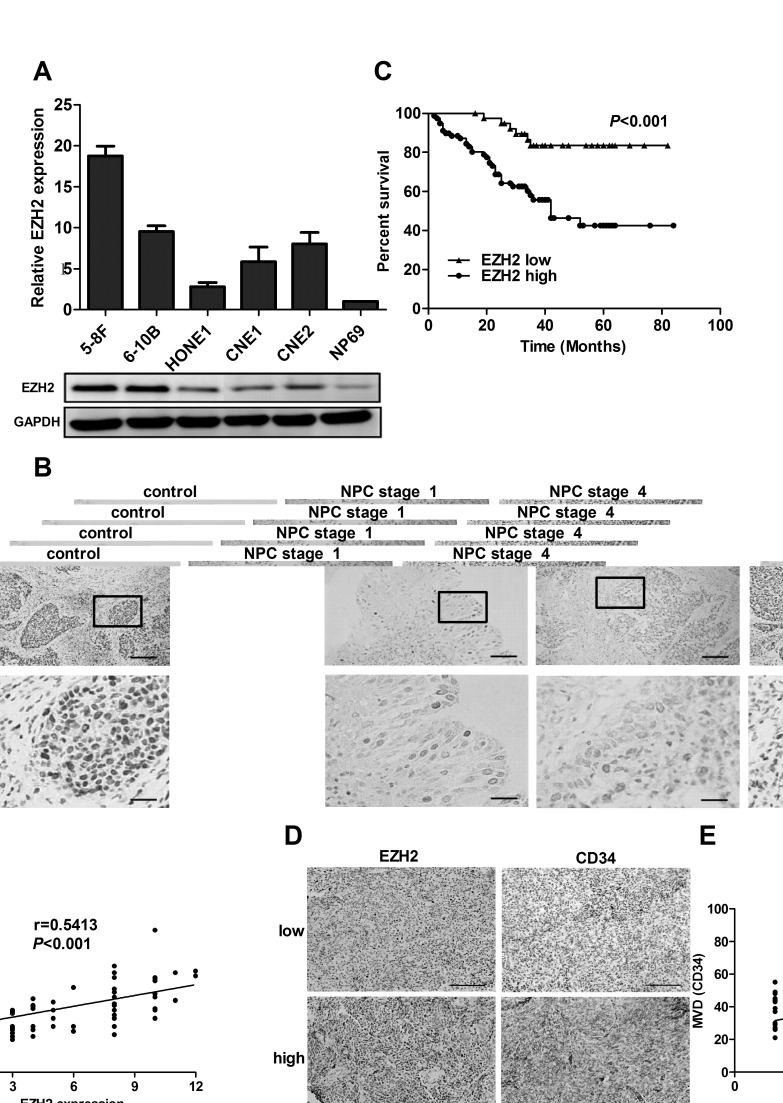
EZH2 was overexpressed and positively correlated with MVD in NPC (A) The mRNA and protein levels of EZH2 in 5 NPC cell lines. (B) Representative pictures of immunohistochemical staining for EZH2 in non-cancer nasopharyngitis biopsy samples and NPC specimens. Scale bars, 100 μm (upper) and 50 μm (lower). (C) Kaplan-Meier curves of disease-specific mortality for patients whose NPC tumors expressed high or low levels of EZH2. (D) Representative images of immunohistochemical staining for MVD with low or high levels of EZH2. Scale bars, 100 μm. (E) A positive correlation between EZH2 expression and MVD in NPC tissues.

We first examined the expression of EZH2 in NPC cell lines. The results showed that both mRNA and protein levels of EZH2 were upregulated in all five NPC cell lines examined, compared with the immortalized nontumorigenic cell line NP69 (Figure 1A). We further evaluated the expression level of EZH2 in 47 non-cancer nasopharyngitis biopsy samples and 135 NPC specimens using immunohistochemical staining. The data showed that EZH2 was highly expressed in NPC samples compared with normal controls (*P*<0.001, Supplementary Figure S1A). Correlation analysis demonstrated that high expression of EZH2 was positively associated with a more advanced clinical stage of NPC (*P*<0.01, Figure 1B and Supplementary Table S1). Survival analysis showed that the mean disease-free survival time for NPC patients with high expression of EZH2 was 30.2 months compared with 56.7 months for patients with low expression of EZH2 (*P*<0.001, Figure 1C). We next evaluated potential associations between EZH2 expression and microvessel density (MVD). Tumors with increased EZH2 expression had significantly greater MVD (*P*<0.001, Figure 1D and Supplementary Figure S1B). When the relative expression level of EZH2 was plotted against that of CD34 in each NPC samples, a significant positive correlation was found (r=0.5413, *P*<0.001, Figure 1E). All these data provided strong evidence that high expression of EZH2 was closely associated with tumor angiogenesis in NPC.

### EZH2 promotes cancer angiogenesis *in vitro* and *in vivo*

**Figure 2:**
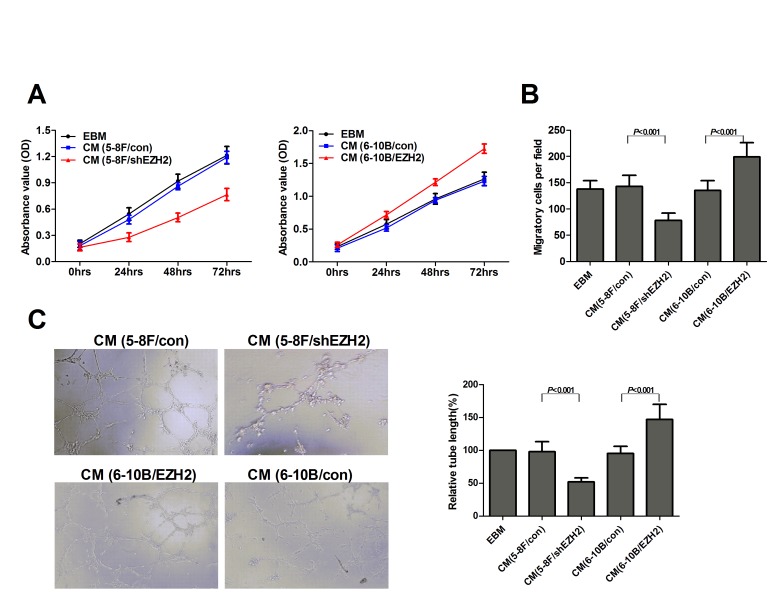
EZH2 enhanced HUVEC proliferation, migration and tubule formation The NPC cell line 5-8F was infected with LV-shEZH2 to downregulate EZH2 expression, and 6-10B was infected with LV-EZH2 to upregulate EZH2 expression. The media were collected as CM and then applied to HUVECs. Then, the cell growth of HUVECs was measured by MTT assay as shown in (A). The cell migration was measured by Transwell migration assay as shown in (B). Tubule formation of HUVECs was examined by *in vitro* tube formation assay as shown in (C). Representative pictures of tubule formation (Left) were taken at 18 hours postplating and quantified for tubule length (Right).

On the basis of our observations from clinical samples, we next asked whether EZH2 could promote angiogenesis in NPC, and elucidated the pro-angiogenic effect of EZH2 with 5-8F and 6-10B cell lines. The 5-8F cell line with high metastatic potential and the 6-10B cell line with no metastatic potential were generated from SUNE-1 cells. In angiogenic processes, endothelial cells must undergo proliferation, migration and tube formation to form new blood vessels [12]. We used MTT, Transwell migration and tube formation assay to measure the angiogenic activity of HUVECs. We found that the conditioned media (CM) from EZH2-upregulated 6-10B cells promoted cell growth, migration and tubule formation of HUVECs (*P*<0.001, Figure 2A-C). In contrast, the CM from EZH2-silencing 5-8F cells inhibited cell growth, migration and tubule formation of endothelial cells (*P*<0.001, Figure 2A-C).

**Figure 3:**
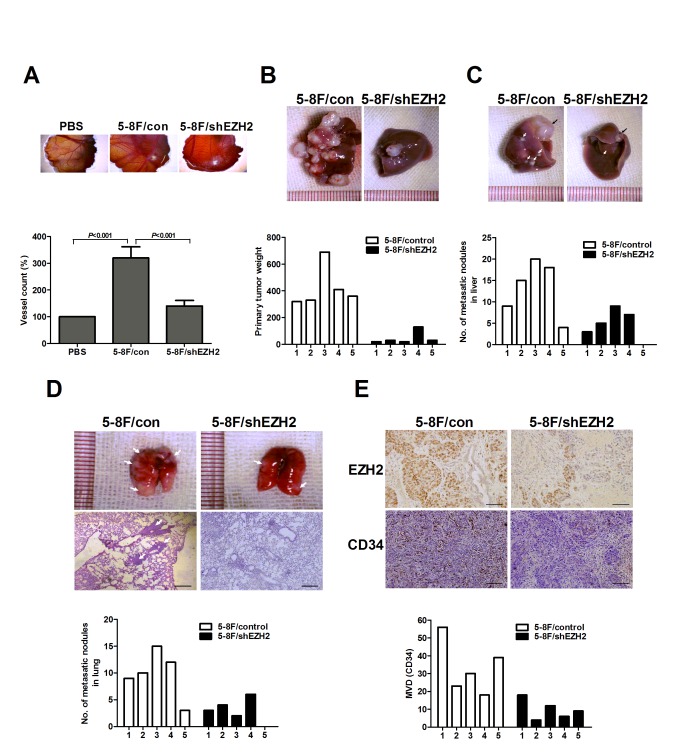
EZH2 promoted *in vivo* angiogenesis and metastasis (A) Chick embryos were incubated with PBS, 5-8F/con CM or 5-8F/shEZH2 CM for six days, and then resected, fixed and photographed with a stereomicroscope. (B) The primary tumor weight of mice injected with LV-shEZH2-infected cells was significantly smaller than that of LV-con-infected 5-8F cells (*P*<0.001). Representative pictures of livers were shown. (C) Representative livers (black arrow, metastatic nodules). Number of metastatic nodules on the surface of the livers of 5-8F/shEZH2 group was significantly less than control group (*P*=0.018). (D) Representative lungs and H&E staining of lung metastatic tumors (White arrow, metastatic nodules). Number of metastatic nodules on the surface of the lungs of 5-8F/shEZH2 group was significantly less than control group (*P*=0.024). (E) Representative images of immunohistochemical staining for EZH2 and MVD in the livers of 5-8F/shEZH2 group or control group. The score of mean MVD in 5-8F/shEZH2 group was significantly higher than that of control group (*P*=0.014). Scale bars, 100 μm.

The *in vitro* results led us to examine the effect of EZH2 on angiogenesis using the *in vivo* model of chick chorioallantoic membrane (CAM) assay. The results showed that CM from 5-8F/shEZH2 inhibited angiogenesis in CAM compared with control (Figure 3A). We also analyzed the pro-angiogenic effect of EZH2 in a murine model of NPC metastasis. Primary tumors were established by direct injection of LV-shEZH2-infected or LV-con-infected 5-8F cells into the liver. Fourteen days postinjection, we sacrificed the mice and dissected the livers and lungs for macroscopic and microscopic histology. The tumors in control group grew more rapidly and attained greater weight than those in 5-8F/shEZH2 group (*P*<0.001, Figure 3B). Livers and lungs of mice bearing EZH2-silencing 5-8F tumors harbored statistically significantly fewer microscopic and macroscopic metastases than those of mice bearing mock-infected 5-8F tumors (*P*<0.001, Figure 3C,). Moreover, tumors with high EZH2 expression in control group had significantly greater MVD than 5-8F/shEZH2 group (*P*<0.001, Figure 3E). Overall, these results indicated that EZH2 promoted angiogenesis and tumor metastasis *in vivo*.

### EZH2 inhibites miR-1 expression in NPC cells

**Figure 4:**
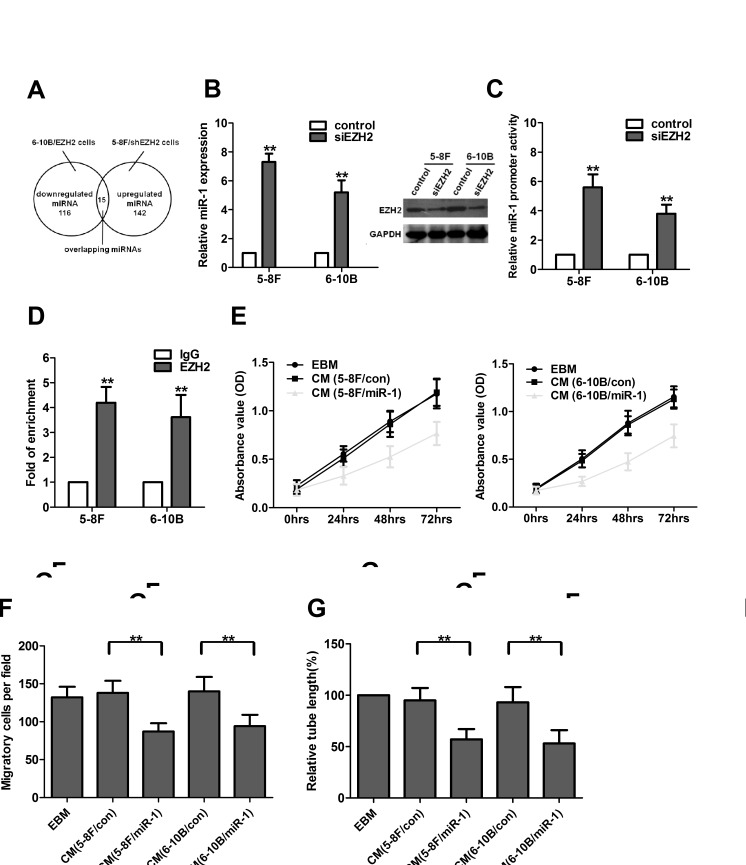
EZH2 inhibited miR-1 expression in NPC cells (A) Fifteen miRNAs were concurrently upregulated in EZH2-silenced 5-8F cells and downregulated in EZH2-overexpressed 6-10B cells. (B) EZH2 expression was significantly downregulated after siEZH2 transfection, and knockdown of EZH2 increased the expression of miR-1 in 5-8F and 6-10B cells. (C) Luciferase activities of miR-1 promoter constructs in 5-8F and 6-10B cells transfected with a negative control or siEZH2. (D) Chromatin from 5-8F and 6-10B cells was immunoprecipitated with an EZH2 antibody or IgG control. Immunoprecipitated DNA was extracted and examined for the presence of miR-1 promoter DNA by qPCR. (E-G) 5-8F and 6-10B cells were infected with LV-miR-1 to upregulate miR-1 expression. The media were collected as CM and then applied to HUVECs. Then, the cell growth, migarion and tubule formation of HUVECs was measured by MTT, Transwell migration and *in vitro* tube formation assay. **, *P*<0.01 compared with control.

To illustrate the unique molecular mechanisms by which EZH2 promoted angiogenesis in NPC, we performed a locked nucleic acid-based human global miR qRT-PCR profiling in 5-8F/shEZH2 and 6-10B/EZH2 cells. Here, 142 (approximately 19%) miRs were upregulated >1.5-fold in EZH2-silenced 5-8F cells. In parallel, 116 (approximately 15%) miRs were downregulated >1.5-fold in EZH2-overexpressed 6-10B cells. When combining both studies, 15 miRs were found both downregulated in EZH2-overexpressed 6-10B cells and upregulated in EZH2-silenced 5-8F cells (Figure 4A, Supplementary Figure S2A). Among these 15 miRNAs, several miRNAs have been confirmed as novel tumor suppressors in regulation of cell growth, angiogenesis and metastasis in different human tumor models, such as miR-502-5p in colon cancer and miR-520c-3p in diffuse large B cell lymphoma [13, 14]. Additionally, miR-718 represses VEGF and inhibits ovarian cancer cell progression, and mediates Nef- and K1-induced angiogenesis via activation of AKT/mTOR signaling in AIDS-Kaposi's sarcoma [15, 16]. In contrast, miR-10b promotes cell migration and invasion in breast cancer [17].

Our data showed that miR-1 had the lowest level in 6-10B/EZH2 cells and the highest level in 5-8F/shEZH2 cells respectively. Additional qRT-PCR validation showed that miR-1 was a promising target because its expression was consistently downregulated in NPC cells and tissues compared with EZH2 upregulation (Supplementary Figure S2B,). Since miR-1 was described earlier as a critical regulator of cardiovascular development [18] and a candidate tumor suppressor in various cancers [19], we focused on miR-1 and investigated the miR-1's contribution to NPC angiogenesis. We further confirmed the miR profiling results by qRT-PCR. In an independent transient EZH2 knockdown experiment, EZH2 expression was significantly downregulated after siEZH2 transfection, and miR-1 expression increased significantly in both 5-8F and 6-10B cells (Figure 4B). To determine whether EZH2 could inhibit miR-1 expression at the promoter level, a non-specific siRNA or siEZH2 along with miR-1 promoter construct were co-transfected into 5-8F and 6-10B cells. Reporter assay showed that EZH2 knockdown significantly increased the promoter activity of miR-1 in both cell lines (Figure 4C). To determine whether EZH2 could bind directly to miR-1 promoter, we performed chromatin immunoprecipitation assay. The results showed that EZH2 enriched miR-1 promoter chromatin by 4.2- and 3.6-fold in both cell lines respectively (Figure 4D). These data demonstrated that EZH2 inhibited miR-1 expression directly through binding to its promoter.

To investigate the functional role of miR-1 in NPC cells, we used lentiviral vectors to stably restore the expression of miR-1 in 5-8F and 6-10B cells, and examined the effect of miR-1 on the angiogenic activity of HUVECs. We found that the CM from miR-1-upregulated NPC cells inhibited cell growth, migration and tubule formation of HUVECs (Figure 4E-G). Additionally, we investigated whether the reduced expression of miR-1 could mimic the pro-angiogenic effect of EZH2. As shown in Supplementary Figure S3A-C, miR-1 knockdown promoted cell growth, migration and tubule formation of HUVECs, similar to those induced by EZH2.

### EZH2 promotes NPC angiogenesis through miR-1-mediated targeting of ET-1

**Figure 5:**
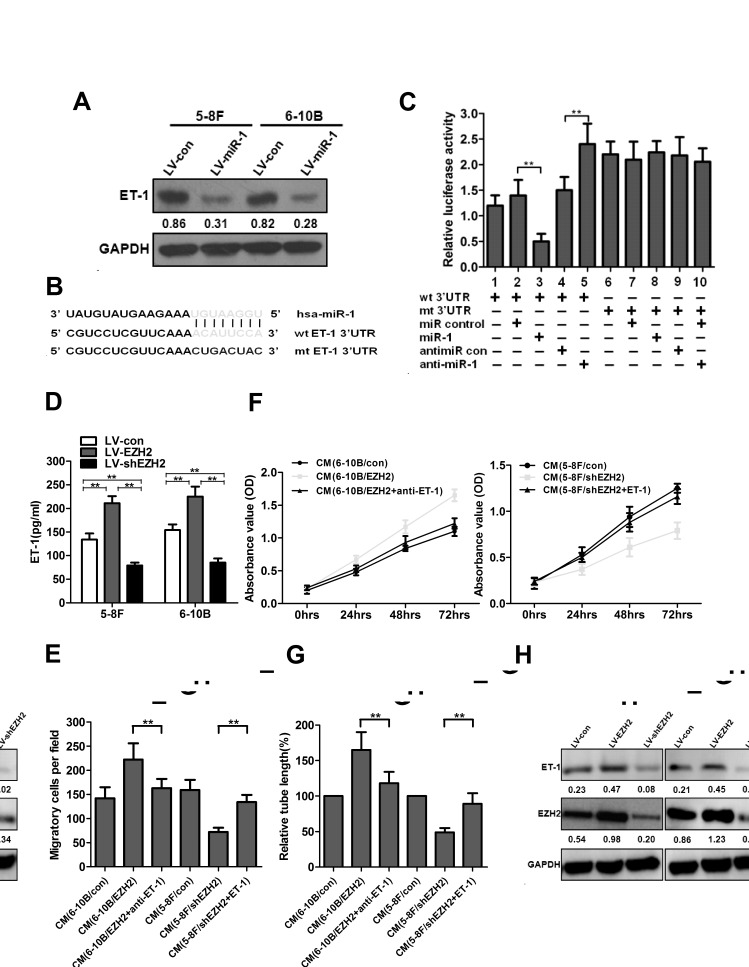
EZH2 promoted NPC angiogenesis through miR-1-mediated targeting of ET-1 (A) The protein levels of ET-1 after LV-miR-1 infection in 5-8F and 6-10B cells. (B) Diagram of ET-1 3′UTR-containing reporter constructs. (C) Luciferase reporter assays in 5-8F cells, with cotransfection of wt or mt 3′UTR and miRNAs as indicated. (D) 5-8F and 6-10B cells were infected with LV-EZH2 or LV-shEZH2, respectively. The concentrations of ET-1 in the conditioned media were measured using an ELISA kit. (E) The cells were treated as indicated in (D). The protein levels of ET-1 in 5-8F and 6-10B cells were examined by Western blot analysis. (F-H) Serum-starved LV-EZH2-infected 6-10B cells were preincubated with a neutralizing antibody against ET-1. EZH2-silenced 5-8F cells were transfected with an ET-1 expression vector to upregulate ET-1 and used for the rescue experiment. The media were collected as CM and then applied to HUVECs. Then, the cell growth, migarion and tubule formation of HUVECs was measured by MTT, Transwell migration and *in vitro* tube formation assay. **, *P*<0.01 compared with control.

To explore the mechanism of miR-1 as an angiogenesis inhibitor, we investigated whether miR-1 could regulate ET-1 expression in NPC cells. ET-1, a novel stimulator of tumor angiogenesis, was reported to be a target of miR-1 in hepatocellular carcinoma [20]. However, our knowledge on ET-1 in NPC tumorigenesis is very limited. We therefore examined ET-1 protein levels in miR-1-overexpressed NPC cells. As shown in Figure 5A, ectopic expression of miR-1 significantly reduced ET-1 protein levels. We further performed luciferase reporter assay to determine whether miR-1 could directly target the 3′UTR of ET-1. The target sequence of ET-1 3′UTR (wt 3′UTR) or the mutant sequence (mt 3′UTR) was cloned into a luciferase reporter vector (Figure 5B). 5-8F cells were then transfected with wt or mt 3′UTR vector and miR-1 mimic. The results showed a significant decrease of luciferase activity when compared with miR control (Figure 5C, lanes 2 and 3; *P*<0.01). The activity of mt 3′UTR vector was unaffected by a simultaneous transfection with miR-1 (Figure 5C, lanes 7 and 8). Moreover, cotransfection with anti-miR-1 and wt 3′UTR vector led to a 1.5-fold increase of luciferase activity (Figure 5C, lanes 4 and 5; *P*<0.01).

To determine whether ET-1 deregulation contributed to EZH2-induced angiogenesis, we established stable ET-1-overexpressed 5-8F and 6-10B cells. We found that ET-1 was upregulated in EZH2-ovexpressed NPC cells, and knockdown of EZH2 reduced the protein level of ET-1, as shown by ELISA and western blot analyses (*P*<0.01, Figure 5D, E). Similar to EZH2 overexpression, ectopic expression of ET-1 significantly promoted cell proliferation, migration and tubule formation of HUVECs (Supplementary Figure S4A-C). Moreover, an ET-1-neutralizing antibody significantly abrogated the pro-angiogenic effect induced by EZH2 overexpression in 6-10B cells (*P*<0.01, Figure 5F-H), and forced expression of ET-1 significantly rescued the anti-angiogenic effect induced by EZH2 knockdown in 5-8F cells (*P*<0.01, Figure 5F-H). All these data indicated that EZH2-induced angiogenesis was mediated by ET-1.

### ET-1 is overexpressed in NPC tissues

**Figure 6:**
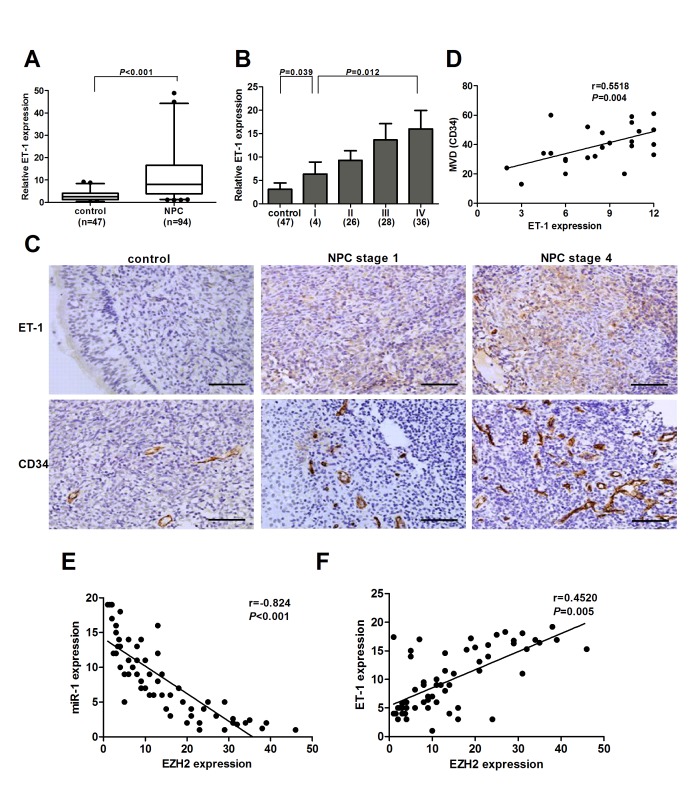
ET-1 was overexpressed in NPC (A) Examination of ET-1 expression in the NPC tissues (n=94) and non-cancer nasopharyngitis tissues (n=47). (B) ET-1 expression decreased in the tissue samples with increasing NPC stage. (C) Representative pictures of immunohistochemical staining for ET-1 and CD34 in NPC specimens and non-cancer nasopharyngitis tissues. Scale bars, 100 μm (upper) and 50 μm (lower). (D) A positive correlation between ET-1 expression and MVD in NPC tissues. (E) An inverse correlation between EZH2 and miR-1 expression in NPC tissues. (F) A positive correlation between ET-1 and EZH2 expression in NPC tissues.

To further determine whether EZH2 expression was associated with ET-1 in NPC tissues, we examined ET-1 expression in 47 non-cancer nasopharyngitis biopsy samples and 94 NPC specimens using qPCR. The results showed that ET-1 was significantly upregulated in NPC tissues as compared with non-cancer nasopharyngitis tissues (*P*<0.001, Figure 6A). ET-1 expression was also increased in NPC cell lines compared with NP69 (Supplementary Figure S5A). In addition, we found that ET-1 expression was higher in stage 4 patients, whereas stage 1-3 had lower levels, showing a significant correlation of ET-1 expression with clinical stages (*P*=0.012, Figure 6B). Immunohistochemical staining of ET-1 in selected tissue samples (30 control and 30 NPC cases) further confirmed the above results (Figure 6C). Notably, tumors with increased ET-1 expression had significantly greater MVD (*P*<0.001, Figure 6C and Supplementary Figure S5B). Correlation analysis demonstrated a significant positive relationship between ET-1 expression and MVD (r=0.5518, *P*=0.004, Figure 6D). In addition, we found that miR-1 and EZH2 had an inverse correlation (Figure 6E) and that EZH2 and ET-1 were positively correlated at the mRNA levels (Figure 6F). These data provided strong evidence that ET-1 was overexpressed in NPC tissues, and its high expression was closely associated with tumor metastasis and angiogenesis.

## DISCUSSION

EZH2, a critical component of the PRC2, has intrinsic histone methyltransferase activity and has been implicated in the progression and metastasis of several cancers [21-23]. The association of EZH2 with the malignant phenotype of multiple solid tumors and its role as a repressor of gene targets lead to the hypothesis that EZH2 could impact specific angiogenic mechanisms of cancer angiogenesis. The first clue on a possible role of EZH2 in tumor angiogenesis came from a report about ovarian carcinoma in 2010 [24], which identified EZH2 as a key regulator of tumor angiogenesis. However, despite the progress that has been made in recent years in this area, our understanding of the molecular mechanisms by which EZH2 contributes to angiogenesis is limited. In this study, we show that EZH2 promotes angiogenesis through inhibition of miR-1/ET-1 axis. To the best of our knowledge, this study is the first to establish that EZH2 is a pro-angiogenic mediator in NPC tumorigenesis and delineate an oncogenic pathway that functionally links EZH2 with ET-1 via miR-1.

Emerging evidence has shown that EZH2 is a key regulator of cancer angiogenesis in ovarian cancer, glioblastoma, Kaposi sarcoma and bladder cancer [24-27]. Our results showed that EZH2 was overexpressed in NPC cell lines and tissues, and its high expression was closely associated with an aggressive and poor prognostic phenotype, which was in consistent with the published report [11]. Notably, we found that high expression of EZH2 was positively correlated with MVD in NPC tissues. Functional studies showed that EZH2 promoted angiogenesis *in vivo* and *in vitro* by stimulating the growth, migration and tubule formation of endothelial cells. All these results suggested that EZH2 played a crucial role in the regulation of NPC angiogenesis.

Recently, some mechanistic insights into EZH2-driven angiogenesis are emerging. Lu *et al*. showed that VEGF stimulation led to upregulated E2F expression, which directly modulated EZH2 levels. EZH2 caused VASH1 silencing by promoter methylation and subsequently promoted angiogenesis in ovarian cancer [24]. In Kaposi sarcoma, KSHV-mediated upregulation of EZH2 was required for the induction of Ephrin-B2, an essential pro-angiogenic factor which drove endothelial cell tube formation [27]. However, we found that no obvious difference of VASH1 or Ephrin-B2 expression was observed between EZH2-overexpressed and EZH2-silenced NPC cells (data not shown), suggesting that the alteration of VASH1 and Ephrin-B2 induced by EZH2 was cell type dependent in cancer cells. In this study, a novel finding was that EZH2 positively regulated ET-1 expression through miR-1, which identified ET-1 as a new target of EZH2 and uncovered a novel molecular mechanism of EZH2-induced angiogenesis. Although several studies have demonstrated EZH2's ability to inhibit miRNA's expression [28-30], the relationship between EZH2 and miR-1 has never been reported. Our results showed that EZH2 inhibited miR-1 expression directly through binding to its promoter.

To investigate the molecular mechanisms by which EZH2 promoted angiogenesis in NPC, we examined the expression and role of miR-1 in NPC tumorigenesis. Our results found that miR-1 was consistently downregulated in NPC cells and tissues, consistent with the data of many types of cancers, such as colorectal cancer, head and neck squamous cell carcinoma and prostate cancer [31-33]. Functional studies showed that forced expression of miR-1 suppressed the growth, migration and tubule formation of endothelial cells, suggesting its role as an anti-angiogenic factor in NPC angiogenesis. Previous studies have shown the aberrant expression of miR-1 in stepwise development of NPC, and confirmed that miR-1 induced apoptosis by targeting PTMA in NPC cells [34, 35]. Therefore, further studies are required to explore the putative tumor-suppressive role of miR-1 in NPC tumorigenesis.

ET-1 acts as a tumorigenic as well as an angiogenic factor [36, 37]. Upregulation of ET-1 has been documented in a wide range of human tumors [38]. Aberrant expression of ET-1, or overexpression of endothelin receptors or their linked signalling circuits can contribute to cell proliferation, apoptosis, migration, epithelial-to-mesenchymal transition, chemoresistance, response of immune cells and neovascularization through both autocrine and paracrine mechanisms [5]. Previous studies have identified ET-1 as a target of miR-1 in hepatocellular carcinoma [20]. In our studies, we confirmed that ET-1 was a direct target of miR-1 in NPC cells. Although the role of ET-1 in cancer is well established, much less is known about the role of ET-1 in NPC tumorigenesis. Wen *et al.* investigated the prognostic role of ET-1 and endothelin A receptor (ETAR) gene polymorphisms in the blood samples of patients with locoregionally advanced NPC, and found that the ETAR/H323H polymorphism was a novel and independent prognostic marker [39]. Another report found that pretreatment plasma big ET-1 levels may be useful in predicting posttreatment distant failure in patients with advanced-stage NPC [40]. However, the status and function of ET-1 have never been documented in NPC. Here, we found that ET-1 was significantly upregulated in NPC tissues, and high expression of ET-1 was closely associated with tumor metastasis and angiogenesis in NPC. Overexpression of ET-1 significantly promoted cell proliferation, migration and tubule formation of endothelial cells, contributing to cancer angiogenesis in NPC. These results identified ET-1 as a new player in NPC angiogenesis. However, the molecular mechanism of ET-1-induced angiogenesis in NPC is still unknown. Further studies are needed to reveal the pathway mediated by ET-1 in NPC angiogenesis.

In summary, we have shown that EZH2 promotes angiogenesis by inhibiting miR-1/ET-1 axis in NPC. These findings provide a better understanding of the mechanisms of cancer angiogenesis in NPC and promising novel targets for anti-angiogenic treatment.

## METHODS

### Cell culture

An immortalized nasopharyngeal epithelial cell NP69 was cultured in Keratinocyte-SFM (Invitrogen, Carlsbad, CA, USA) supplemented with bovine pituitary extract (BD Biosciences, Bedford, MA, USA). The human NPC cell lines 5-8F, 6-10B, CNE1, CNE2 and HONE1 were cultured in RPMI-1640 (Invitrogen). HEK 293T cells were cultured in Dulbecco's modified Eagle's medium (DMEM; Invitrogen). Human umbilical vein endothelial cells HUVECs were cultured in endothelial cell basal medium (EBM, PromoCell, Heidelberg, Germany).

### Clinical samples

The NPC biopsy specimens (n=135) and non-cancer nasopharyngitis biopsy samples (n=47) were collected from Nanfang Hospital between January 2004 to December 2008. All biopsy samples were pathologically reassessed. There were 94 NPC patients with a long time follow-up up to 100 months. None of the patients had received radiotherapy or chemotherapy before biopsy sampling. TNM stage designation was according to the definitions of the seventh edition of the UICC-American Joint Committee on Cancer staging criteria. The clinicopathological characteristics of NPC patients were summarized in Supplementary Table S1. Informed consent was obtained from all individuals, and the research protocols were approved by the Ethics Committee of Nanfang Hospital.

### Plasmids, transfection and lentivirus transduction

A 191 bp DNA fragment corresponding to pre-miR-1-1 and the flanking sequence was amplified from human genomic DNA and then cloned into pLVTHM lentiviral vector (http://www.addgene.org/Didier_Trono). Two lentiviral vectors for cDNA and shRNA delivery of EZH2 were described previously [41, 42]. The production, purification and titration of lentivirus were performed as described by Tiscornia *et al* [43]. The packaged lentiviruses were named LV-miR-1, LV-EZH2 and LV-shEZH2 respectively. The empty lentiviral vector LV-con was used as a control. The recombinant vector for cDNA delivery of ET-1 was purchased from GeneCopoeia. EZH2 siRNA or scramble control siRNA was purchased from Applied Biosystems (Foster City, CA, USA). Transient transfection of miR-1 mimic or siRNA was performed using Lipofectamine 2000 reagent (Invitrogen).

### RNA isolation, reverse transcription, and quantitative real-time PCR

MicroRNA profilings were conducted using microRNA Ready-to-Use PCR, Human panel I+II, V3.M (Exiqon, Vedbaek, Denmark). Total RNA was extracted using Trizol reagent (Invitrogen). To quantitate miR-1 expression, total RNA was polyadenylated and reversely transcribed using NCode miRNA First-Strand cDNA Synthesis kit (Invitrogen). To measure the mRNA levels of EZH2 and ET-1, total RNA was reversely transcribed using ImProm-II Reverse Transcription System (Promega, Madison, WI, USA). Quantitative real-time PCR (qPCR) was performed using SYBR Green PCR master mix (Applied Biosystems) on an ABI 7900HT System. The primers were listed in Supplementary Table S2. GAPDH or U6 snRNA was used as an endogenous control. All samples were normalized to internal controls and fold changes were calculated through relative quantification (2^−ΔΔCt^; ref. 44).

### Western Blot analysis

Western blot analysis was carried out using antibodies to EZH2 (Cell Signaling Technology, Danvers, MA, USA) and ET-1 (Santa Cruz Biotechnology, Santa Cruz, CA, USA). GAPDH was used as a protein loading control. Immunoreactive bands were visualized by autoradiography following development with an enhanced chemiluminescence system (GE Healthcare Life Sciences, Piscataway, NJ, USA).

### Evaluation of ET-1 secretion

ET-1 concentrations in cell culture supernatant were measured with a Quantikine ELISA kit (R&D Systems, Minneapolis, MN, USA). All standards and samples were prepared and tested in triplicate according to the manufacturer's instructions and the mean value taken.

### Immunohistochemistry (IHC)

Immunohischemical analysis of EZH2 protein was performed as described previously [11]. Rabbit polyclonal anti-EZH2 antibody (Cell Signaling Technology) was diluted at 1:100. IHC staining of ET-1 was performed as described by Wu *et al* [45]. Goat polyclonal anti-ET-1 antibody was diluted at 1:50. The results were scored by intensity (0-3) and percentage of positive cells (0-4). We multiplitied the intensity by percentage score as the final scores (0-12). The cut-point for high expression of EZH2 was defined to be above the median value. For quantification of microvessel density (MVD) in the human NPC and mouse tumor samples, the number of blood vessels staining positive for CD34 (1:100 dilution, Abcam, Cambridge, MA, USA) was recorded in ten random fields at 200 magnification.

### Promoter and 3′UTR Luciferase Reporter Assays

Promoter region of pre-miR-1-1 (ch20q13.2-13.3), 2.2 kb upstream of transcriptional start site, was cloned into pGL3 vector (Promega). A 1103 bp fragment of ET-1 3′UTR was amplified by PCR and cloned downstream of the firefly luciferase gene in pGL3 vector. The vector was named wild-type (wt) 3′UTR. Site-directed mutagenesis of the miR-1 binding site in ET-1 3′UTR was performed using GeneTailor Site-Directed Mutagenesis System (Invitrogen) and was named mutant (mt) 3′UTR. The primers sequences were listed in Supplementary Table S2. For the promoter activity assay, empty pGL3 vector and pGL3-miR-1 promoter were co-transfected with a scrambled siRNA control or an EZH2 siRNA into NPC cells. The pRL vector was used as an internal control. Luciferase activity was measured 48 hours after transfection using the Dual-Luciferase Reporter Assay System (Promega). For reporter assays, wt or mt 3′UTR vector and the control vector pRL were co-transfected.

### Chromatin Immunoprecipitation assay

Chromatin immunoprecipitation (ChIP) was performed using the Chromatin Immunoprecipitation assay kit (Millipore, Billerica, MA, USA) according to the manufacturer's instructions. DNA immunoprecipitated by anti-EZH2 antibody was purified. The DNA was then extracted with phenol-chloroform and precipitated with ethanol. Real-time PCR using specific primers (Supplementary Table S2) to amplify the pre-miR-1-1 genomic loci was carried out in the immunoprecipitated DNA. The enrichment of EZH2 on miR-1 promoter locus was calculated as the fold increase of the immunoprecipitated DNA compared with IgG-precipitated DNA.

### *In vitro* assays

For conditioned media (CM) studies, HUVECs were incubated with EBM alone (Control), or conditioned media obtained from: LV-con infected 5-8F cells (5-8F/con), LV-shEZH2 infected 5-8F cells (5-8F/shEZH2), LV-con infected 6-10B cells (6-10B/con), LV-EZH2 infected 6-10B cells (6-10B/EZH2), LV-miR-1 infected 5-8F cells (5-8F/miR-1), LV-miR-1 infected 6-10B cells (6-10B/miR-1). Cells were incubated for 96 hours to measure migration, proliferation and tube formation. Cell migration and viability was determined by Transwell migration assays and MTT assay as described previously [25, 46]. Tube formation assay was performed as described previously [45].

### Chick chorioallantoic membrane (CAM) assay

Angiogenic activity was examined using a CAM assay as described previously [26]. Fertilized chicken eggs were incubated at 37°C. On day 6, CM from 5-8F/control or 5-8F/shEZH2 cells (2×10^6^ cells) were deposited in the center of the chorioallantoic. The assay was scored and photographed on the 12th embryonic day. PBS was used as a negative control. Chorioallantoid membranes were collected for microscopy and photographic documentation. Angiogenesis was quantified by counting the number of blood vessel branch, and ten viable embryos were tested for each treatment. All animal experiments were carried out in accordance with a protocol approved by Institutional Animal Care and Use Committee of Southern Medical University.

### Animal studies

For tumor metastasis assay *in vivo*, we used a murine model of NPC metastasis successfully established previously [47]. To establish this model, we inoculated NPC cells into the liver as a single nodule which would subsequently metastasized to other parts of the liver and the lung. A total of 10 nude mice were randomly divided into two groups (five mice per group): (a) injection of 5-8F/con cells; (b) injection of 5-8F/shEZH2 cells. The mice were sacrificed and autopsied on day 14, and the growth of primary xenografted tumor and the incidence of lung or liver metastasis were recorded.

### Statistical analysis

SPSS 13.0 software was used for statistical analysis. Data were presented as Mean±SEM of at least three independent experiments. Two-tailed Student's *t* test was used for comparisons of two independent groups. Comparisons of multiple independent groups were analyzed using One-way ANOVA followed by a Student-Newman-Keuls test. The relationships between CD34 expression and EZH2 or ET-1 and the relationships between EZH2 and miR-1 or ET-1 expression were explored by Spearman's correlation. Significant associations between EZH2 expression and clinicopathological parameters were assessed by a chi-squared test. Survival analysis was evaluated by Kaplan-Meire survival plot followed by a log-rank test. *P* values of <0.05 were considered statistically significant.

## SUPPLEMENTARY MATERIAL, FIGURES AND TABLES


