# Supplementary material for: EZH2 promotes angiogenesis through inhibition of miR-1/Endothelin-1 axis in nasopharyngeal carcinoma
Source: Oncotarget. 2014 Sep 3;5(22):11319–32. doi: 10.18632/oncotarget.2435 (PMC4294357; doi:10.18632/oncotarget.2435)
Supplement: Supplementary file 1 [file oncotarget-05-11319-s001.pdf]

EZH2 promotes angiogenesis through inhibition of miR-1/Endothelin-1 axis in nasopharyngeal carcinoma

Supplementary Material

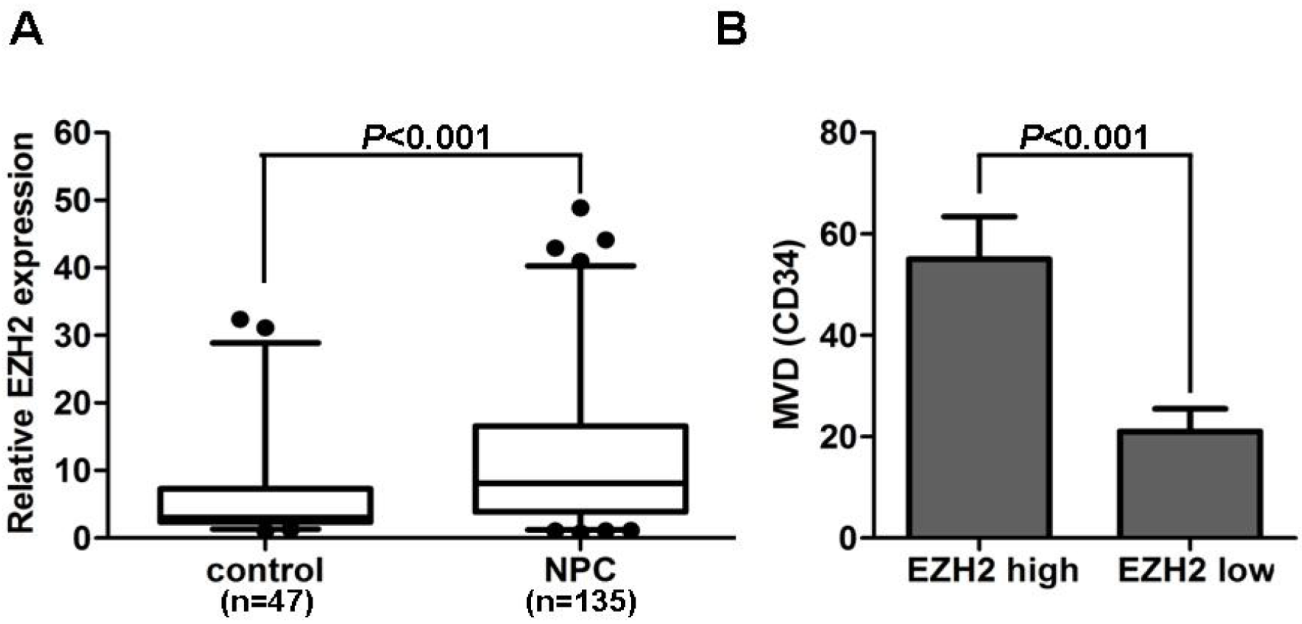

Supplementary Figure 1: EZH2 expression in NPC tissues. **(A)** Relative expression of EZH2 in non-cancer nasopharyngitis biopsy samples (n=47) and NPC specimens (n=135). **(B)** Differences in mean MVD based on EZH2 expression levels in human NPC tissues.

A

	6-10B/EZH2 cells	5-8F/shEZH2 cells
hsa-miR-1	-9.21	6.24
hsa-miR-10b	-1.87	3.02
hsa-miR-24	-4.11	4.56
hsa-miR-181a-2*	-1.55	1.71
hsa-miR-210	-6.10	4.69
hsa-miR-302c*	-5.04	3.29
hsa-miR-342-5p	-1.48	1.59
hsa-miR-502-5p	-1.69	1.93
hsa-miR-520c-3p	-2.44	2.05
hsa-miR-593*	-1.35	1.88
hsa-miR-658	-2.13	2.07
hsa-miR-718	-3.75	4.42
hsa-miR-1247	-2.52	2.75
hsa-miR-1539	-4.93	2.33
hsa-miR-1909*	-3.49	3.27

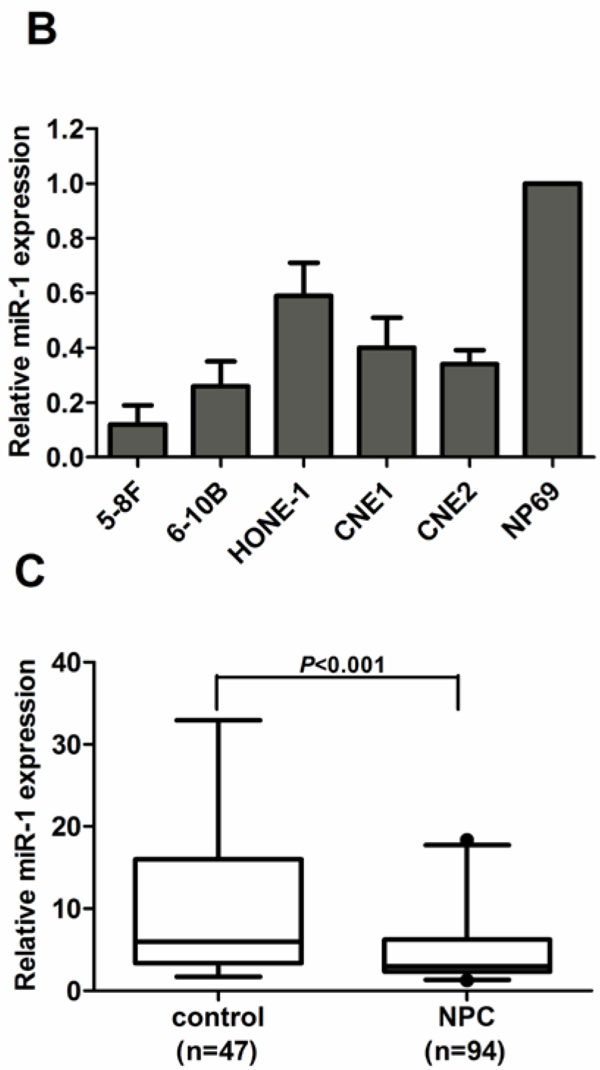

Supplementary Figure 2: Relative expression of miR-1 in NPC cells and tissues. **(A)** By qRT-PCR-based global human miRNA profiling, 15 miRNAs were both upregulated in EZH2-silenced 5-8F cells and downregulated in EZH2-overexpressed 6-10B cells. Fold change was measured by comparative CT method, and change >1.5-fold was considered significant. **(B)** Relative expression of miR-1 in 5 NPC cell lines. **(C)** Examination of miR-1 expression in the NPC tissues (n=94) and non-cancer nasopharyngitis tissues (n=47).

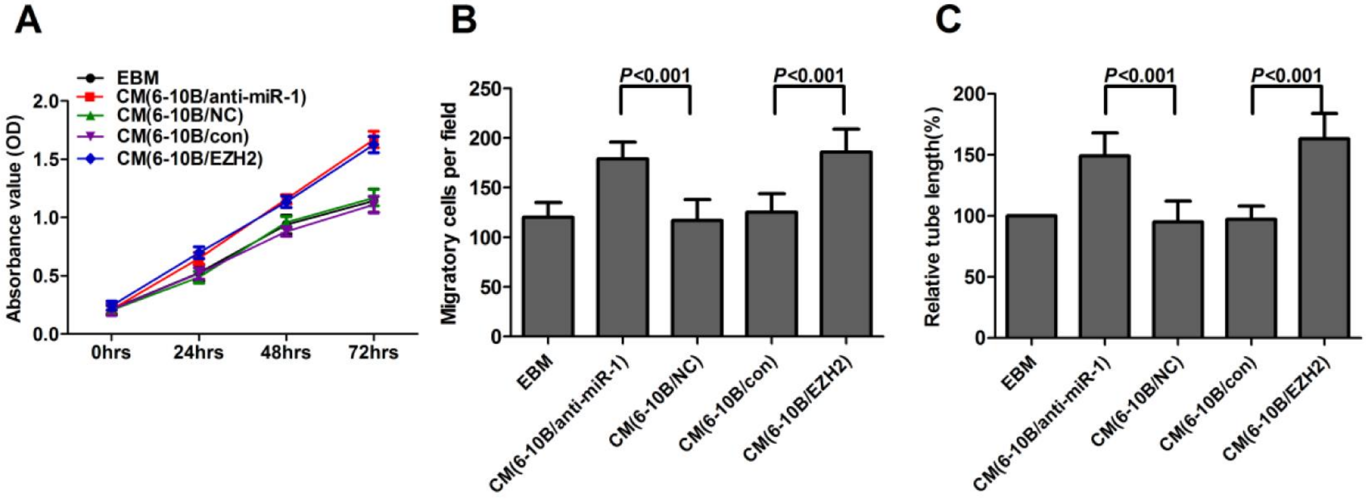

Supplementary Figure 3: The effect of miR-1 on cell growth, migration and tube formation of HUVEC cells. The NPC cell line 6-10B was transfected with LV-EZH2 and miR-1 inhibitor respectively. The media were collected as CM and then applied to HUVECs for 96 hours. Then, the cell growth of HUVECs was measured by MTT assay as shown in **(A)**. The cell migration was measured by Transwell migration assay as shown in **(B).** Tubule formation of HUVECs was examined by *in vitro* tube formation assay as shown in **(C)**.

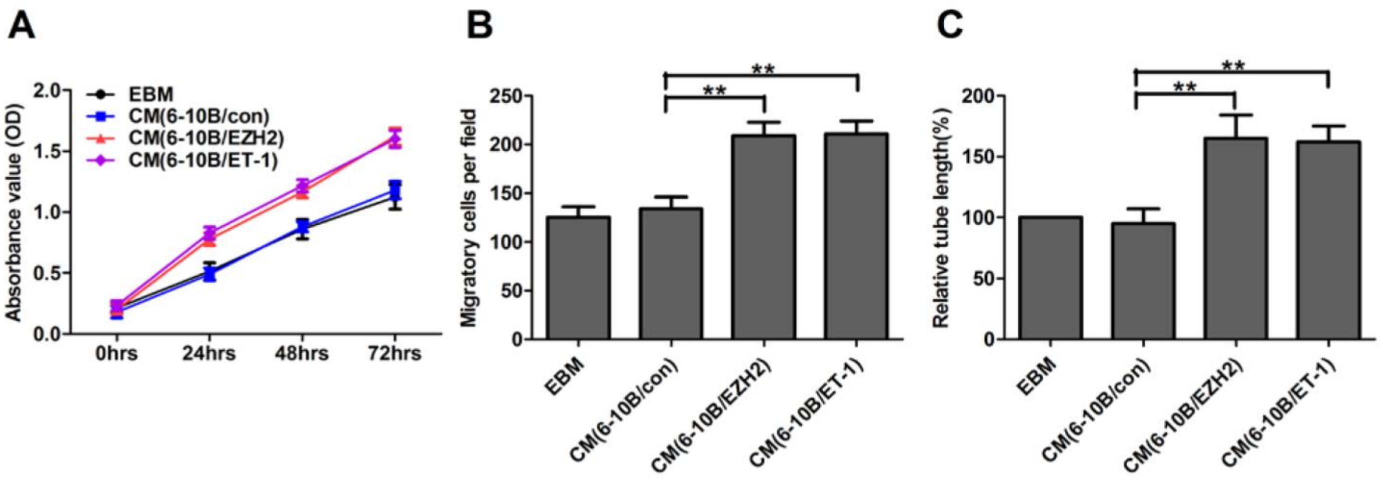

Supplementary Figure 4: Ectopic expression of ET-1 promoted HUVEC proliferation, migration and tubule formation. The NPC cell line 5-8F was infected with LV-EZH2 or LV-ET-1 to upregulate EZH2 and ET-1 expression, respectively. The media were collected as CM and then applied to HEVUCs for 96 hours. Then, the cell growth of HUVECs was measured by MTT assay as shown in **(A)**. Tubule formation of HUVECs was examined by *in vitro* tube formation assay as shown in **(B)**. The cell migration was measured by Transwell migration assay as shown in **(C)**. **, $P<0.01$.

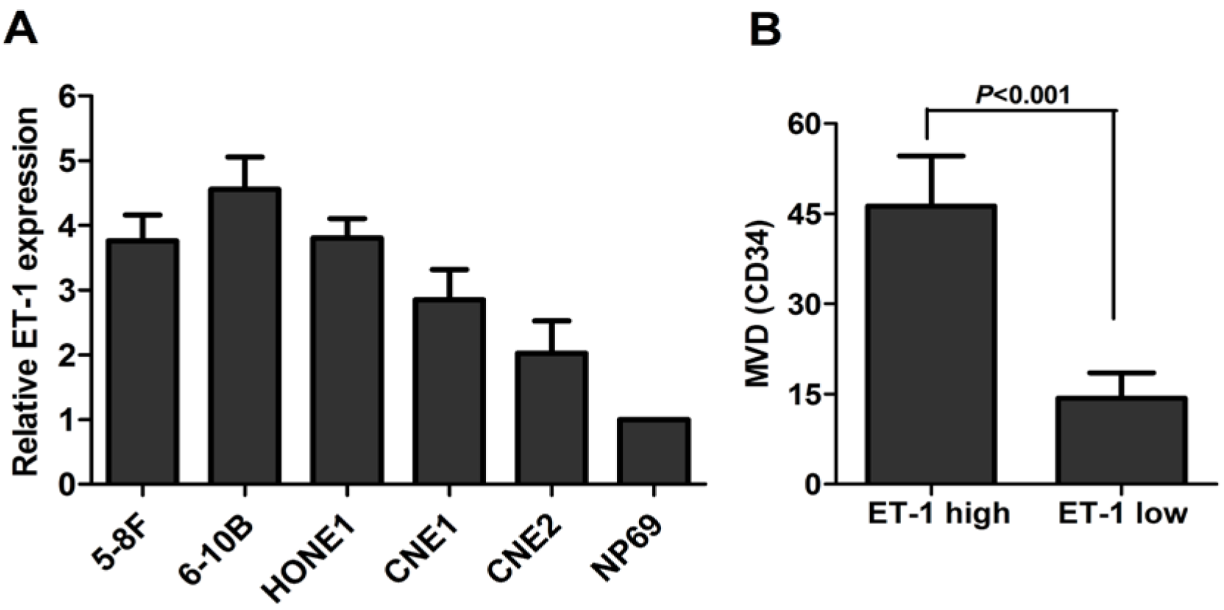

Supplementary Figure 5: ET-1 expression in NPC cells. **(A)** Relative expression of ET-1 in 5 NPC cell lines. **(B)** Differences in mean MVD based on ET-1 expression levels in human NPC tissues.

Supplementary Table S1: Relationship between EZH2 expression and clinicopathologic parameters in 135 NPC patients.

Variable	No.	EZH2, %		*P*
		High (n=90)	Low (n=45)	
Age, year				0.296
<45	92	64 (69.6%)	28 (30.4%)	
≥45	43	26 (60.5%)	17 (39.5%)	
Gender				0.699
Male	90	61 (67.8%)	29 (32.2%)	
Female	45	29 (64.4%)	16 (35.6%)	
T status				0.003
T1	16	6 (37.5%)	10 (62.5%)	
T2	59	35 (59.3%)	24 (40.7%)	
T3	25	20 (80.0%)	5 (20.0%)	
T4	35	29 (82.9%)	6 (17.1%)	
N status				<0.001
N0	37	14 (37.8%)	23 (62.2%)	
N1	38	25 (65.8%)	13 (34.2%)	
N2	36	31 (86.1%)	5 (13.9%)	
N3	24	20 (83.3%)	4 (16.7%)	
M status				<0.001
M0	121	77 (63.6%)	44 (36.4%)	
M1	14	13 (92.9%)	1 (7.1%)	
Stage				<0.001
I	6	1 (16.7%)	5 (83.3%)	
II	37	15 (40.5%)	22 (59.5%)	
III	31	24 (77.4%)	7 (22.6%)	
IV	61	50 (82.0%)	11 (18.0%)	

Supplementary Table S2: Primers used in this study.

Primer name	Sequence (5'-3')
EZH2 qPCR F	GCCAGACTGGGAAGAAATCTG
EZH2 qPCR R	TGTGTTGGAAAATCCAAGTCA
ET-1 qPCR F	CCAAGCTTGGAACAGTCTTTTCCT
ET-1 qPCR R	GGACATCATTTGGGTCAACACTCC
GAPDH qPCR F	GCACCGTCAAGGCTGAGAAC
GAPDH qPCR R	TGGTGAAGACGCCAGTGGA
miR-1 promoter F	TAAAGTGGGGACAGCAAAATGC
miR-1 promoter R	AGCACAAGGTAGAGAAGGTAGAG
miR-1 promoter ChIP F	AACCACAAGGGGATGCCTTC
miR-1 promoter ChIP R	AGAGACCAGAGGCCTGAACT
ET-1 3'UTR F	CAGACCTTCGGGGCCTGT
ET-1 3'UTR R	TTTCTAAAGTCATTACCTTGACAGGCA
Mutant ET-1 3'UTR F	GGACCAGCGTCCTCGTTCAAAAACTCTACAGAAAGGTTAAGGAGTTCCC
Mutant ET-1 3'UTR R	GGGAACTCCTTAACCTTTCTGTAGAGTTTTTGAACGAGGACGCTGGTCC
